# Supplementary material for: Netrin‐1 and its receptor DCC modulate survival and death of dopamine neurons and Parkinson’s disease features
Source: EMBO J. 2020 Dec 22;40(3):e105537. doi: 10.15252/embj.2020105537 (PMC7849168; doi:10.15252/embj.2020105537)
Supplement: Supplementary file 1 — Appendix [file EMBJ-40-e105537-s001.pdf]

Appendix

Table of contents (TOC)

Appendix Figure S1 – page 2

Appendix Figure S2 – page 4

Appendix Figure S3 – page 6

Appendix Figure S4 – page 8

Appendix Figure S1. Netrin-1 depletion induces dopamine neuronal cell death and upregulates DCC and UNC5B receptors (Related to Figure 1 and 2)

A. Immunostainings of DCC or UNC5B in the rat SN region. DCC and UNC5B (red), Tyrosine hydroxylase (TH) (green), DAPI (blue).

B. Immunofluorescent staining of netrin-1 and DCC on nigral sections from netrin-1$^{fl/fl}$ mice. Netrin-1 (Cy5), DCC (red), DAPI (blue) (Scale bar: 500 µm).

C. Immunofluorescent staining of netrin-1 and UNC5B on nigral sections from netrin-1$^{fl/fl}$ mice. Netrin-1 (Cy5), UNC5B (red), DAPI (blue) (Scale bar: 500 µm).

D. Immunofluorescent staining of TH and VMAT2 on netrin-1$^{fl/fl}$ mice brain sections injected with control adenovirus vector. TH (red) and VMAT2 (Cy5) (Scale bar: 500 µm). Quantification of TH and VMAT2 fluorescent intensity in SN and striatum (Str). N = 3 each group. Error bars represent the mean ± SEM. Statistical significance was determined using an unpaired t-test.

E. Immunofluorescent staining of TH and VMAT2 on netrin-1$^{fl/fl}$ mice brain sections injected with Cre adenovirus vector. TH (red) and VMAT2 (Cy5) (Scale bar: 500 µm). Quantification of TH and VMAT2 fluorescent intensity in SN and striatum (Str). N = 3 each group. Error bars represent the mean ± SEM. Statistical significance was determined using an unpaired t-test.

Appendix Figure S2. Netrin-1 titration induces primary neuron death and DCC/UNC5B cleavage by caspases

A-B. Primary cortical **(A)** and dopamine **(B)** neurons (DIV 10) were treated with 100 – 200 ng of human recombinant netrin-1 protein, or a titrating netrin-1 mAb (2F5) 0.5- 5 μg and a recombinant netrin-1 binding protein (4Fbn, the 4th fibronectin domain of the extracellular domain of DCC) 0.5-5 μg for 24 hr. Immunoblot showed the cleaved UNC5B, DCC and caspase 3 after 2F5 and 4Fbn incubation.

C. LDH assay on primary cortical neurons and dopamine neurons (DIV 10). Data are shown as mean ± SEM. Statistical significance was determined by an unpaired t-test. N = 3 each group. *P< 0.05, **P< 0.01.

D. Immunoblot from primary midbrain dopamine neurons treated with 3 caspase 3 or pan-caspase inhibitor. (E-F) TUNEL assay in primary neurons with knockdown of DCC or UNC5B.

E-F. TUNEL assay on primary cortical neurons (DIV 10) treated with shDCC or shUNC5B and control adenovirus vector for 24 hr. **(E)** Representative images. **(F)** Quantification bar graph. Data are shown as mean ± SEM. Statistical significance was determined by an unpaired t-test. N = 3 each group. *P< 0.05, **P< 0.01. All experiments were repeated 3 times.

Appendix Figure S3. Netrin-1 is neuroprotective in a progressive and an acute mouse model of PD

A. Validation of tamoxifen-induced netrin-1 overexpression (O/E) in netrin-1 inducible transgenic mice, by western blot (bottom panel). The spinal cord (sc) of E11.5 mice embryo was used as a positive control. N=4 brains/condition.

B. Representative images by immunohistochemistry of S129-phosphorylated alpha synuclein in wild type (WT) and SNCA transgenic (SNCA Tg) mice (Scale bar, 100 μm). Quantification bar graph of S129-phosphorylated alpha synuclein-positive cell count (right panel). Data are shown as mean ± SEM. Statistical significance was determined by an unpaired t-test. N = 3 each group. *P< 0.05, **P< 0.01.

Appendix Figure S4. Characterization of netrin-1 distribution and effects after intrastriatal injection

A-B. Distribution of 125I-netrin-1 and 125I-GDNF after intrastriatal injection in rats. **(A)** Radioactivity of 125I-labelled proteins in different rat brain areas. Means +SEM are shown, N=10 animals in each group. **(B)** Photomicrograph of an autoradiographic film after intrastriatal injections of 125I-netrin-1 and 125I-GDNF in rats. Brain slice figures modified from the rat brain atlas of Paxinos and Watson (1997). N=10 animals in each 4 group.

C. Diffusion of netrin-1 24h after intrastriatal injection in rats. Fluorescence microscopy representative images of netrin-1 His-tagged (Red) in cortex (CTX), striatum (STR), hippocampus (HIPPO) and substantia nigra (SN); "C": control side, "L" : Lesioned side, Green : TH staining for dopamine (and catecholaminergic neurons) (Scale bar, 2000 μm). N=4 animals/group.

D. Quantification of TH positive neurons from mouse (E13) ventral midbrain primary culture treated with recombinant human Netrin-1, GDNF (positive control) or no factor (negative control). Means + SEM are shown, N = 4. Unpaired t-test, $*P < 0.05$, $**P < 0.01$ compared to control group.

E. Measurement of TH+ surface area normalised by the number of total TH+ cell from mouse (E13) ventral midbrain primary culture. Unpaired t-test $*P < 0.05$, N=3 independent experiments, Means + SEM are shown

F. Immunoblot on striatal rat lysates showing the modulation of DCC, PS6 (Ser235/236), PTEN levels 4h after intrastriatal injection of recombinant human Netrin-1. N=3 animal/condition.

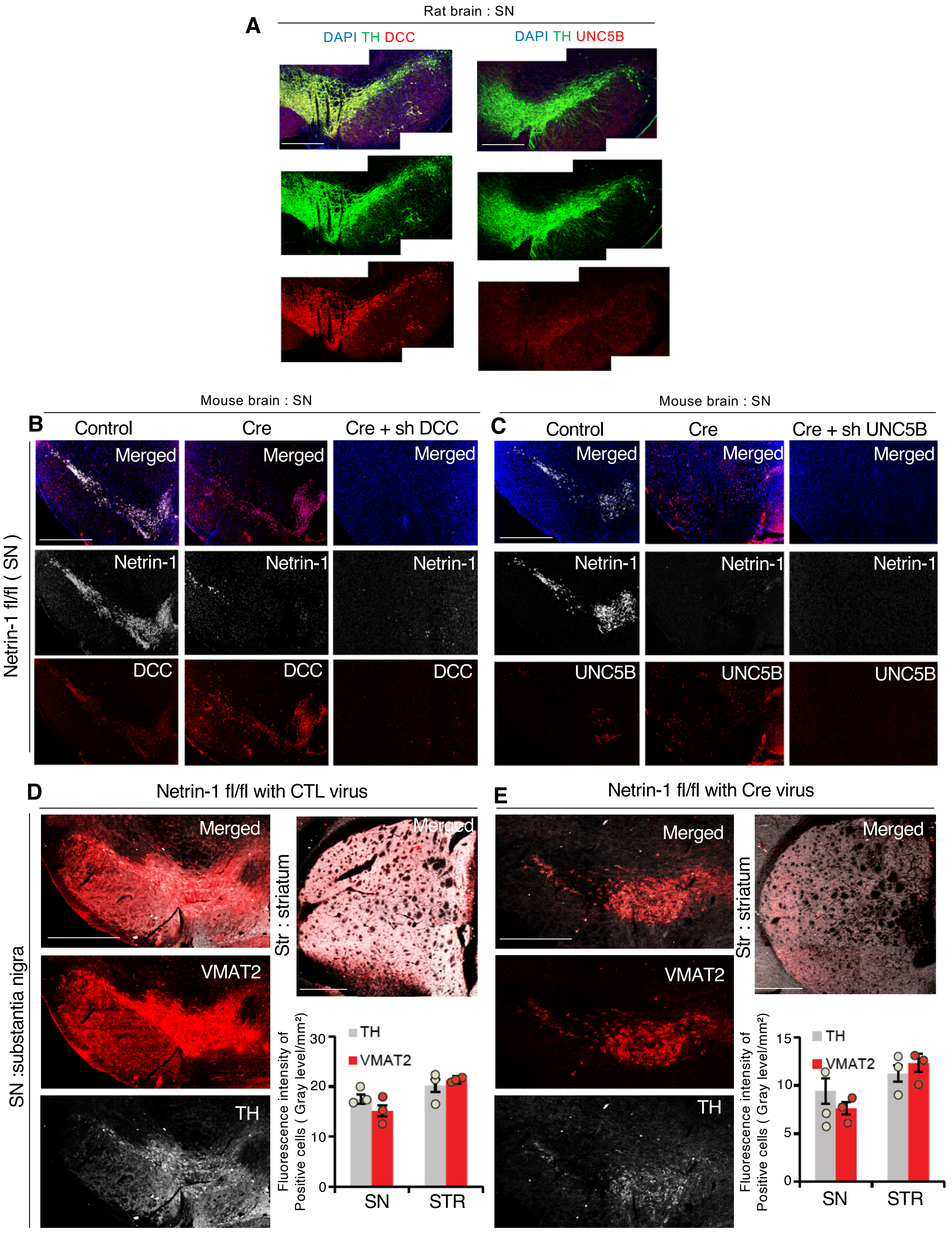
A
Rat brain : SN
DAPI TH DCC
DAPI TH UNC5B
B
Mouse brain : SN
Control
Cre
Cre + sh DCC
Netrin-1 fl/fl (SN)
Merged
Netrin-1
DCC
C
Mouse brain : SN
Control
Cre
Cre + sh UNC5B
Merged
Netrin-1
UNC5B
D
Netrin-1 fl/fl with CTL virus
SN :substantia nigra
Merged
VMAT2
TH
Str : striatum
Merged
Fluorescence intensity of Positive cells (Gray level/mm²)
TH
VMAT2
0
10
20
30
SN
STR
E
Netrin-1 fl/fl with Cre virus
Merged
VMAT2
TH
Str : striatum
Merged
Fluorescence intensity of Positive cells (Gray level/mm²)
TH
VMAT2
0
5
10
15
SN
STR

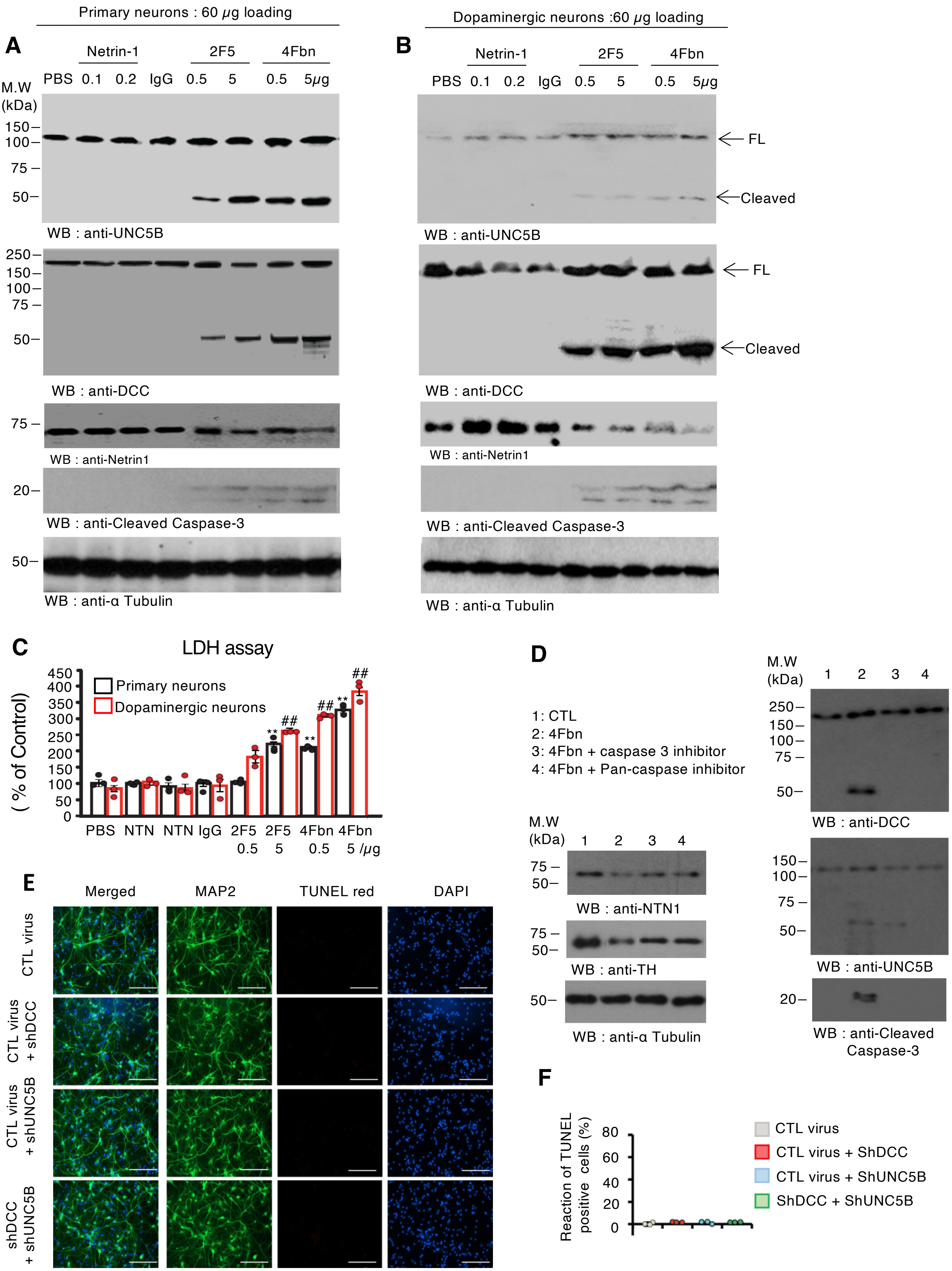

Primary neurons : 60 μg loading
A
Netrin-1
2F5
4Fbn
PBS 0.1 0.2 IgG 0.5 5 0.5 5μg
M.W (kDa)
WB : anti-UNC5B
WB : anti-DCC
WB : anti-Netrin1
WB : anti-Cleaved Caspase-3
WB : anti-α Tubulin
Dopaminergic neurons :60 μg loading
B
FL
Cleaved
C
LDH assay
Primary neurons
Dopaminergic neurons
(% of Control)
PBS NTN NTN IgG 2F5 2F5 4Fbn 4Fbn
0.5 5 0.5 5 /μg
D
1: CTL
2: 4Fbn
3: 4Fbn + caspase 3 inhibitor
4: 4Fbn + Pan-caspase inhibitor
WB : anti-NTN1
WB : anti-TH
WB : anti-Cleaved Caspase-3
E
Merged
MAP2
TUNEL red
DAPI
CTL virus
CTL virus + shDCC
CTL virus + shUNC5B
shDCC + shUNC5B
F
Reaction of TUNEL positive cells (%)
CTL virus
CTL virus + ShDCC
CTL virus + ShUNC5B
ShDCC + ShUNC5B

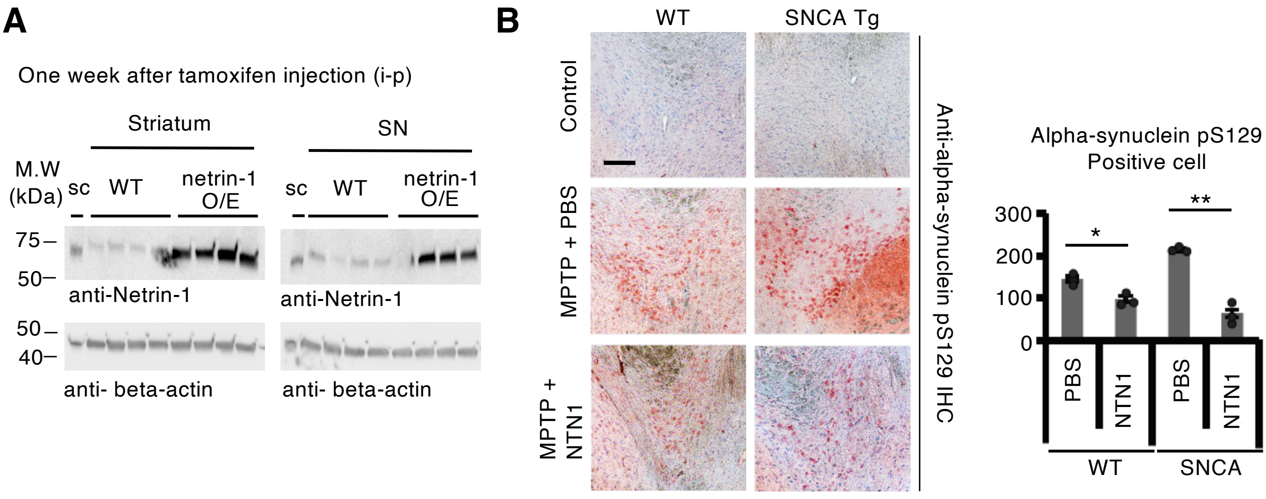
A
One week after tamoxifen injection (i-p)
Striatum
SN
M.W
(kDa)
sc
WT
netrin-1
O/E
75
50
anti-Netrin-1
50
40
anti- beta-actin
B
WT
SNCA Tg
Control
MPTP + PBS
MPTP +
NTN1
Anti-alpha-synuclein pS129 IHC
Alpha-synuclein pS129
Positive cell
300
200
100
0
*
**
PBS
NTN1
WT
SNCA

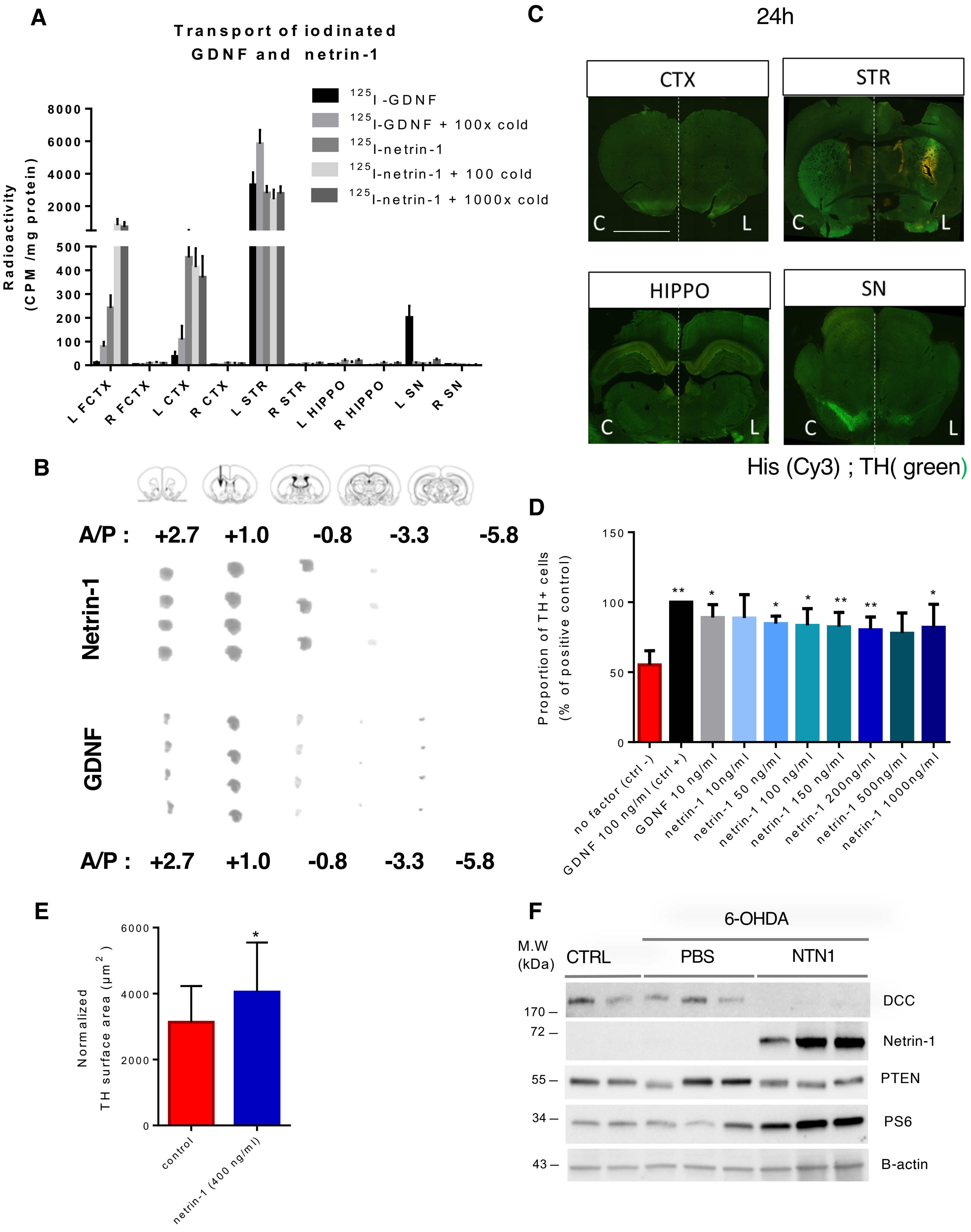
A
Transport of iodinated GDNF and netrin-1
125I -GDNF
125I-GDNF + 100x cold
125I-netrin-1
125I-netrin-1 + 100 cold
125I-netrin-1 + 1000x cold
Radioactivity (CPM /mg protein)
L FCTX
R FCTX
L CTX
R CTX
L STR
R STR
L HIPPO
R HIPPO
L SN
R SN
B
A/P : +2.7 +1.0 -0.8 -3.3 -5.8
Netrin-1
GDNF
C
24h
CTX
STR
HIPPO
SN
His (Cy3) ; TH(green)
D
Proportion of TH+ cells (% of positive control)
no factor (ctrl -)
GDNF 100 ng/ml (ctrl +)
GDNF 10 ng/ml
netrin-1 10ng/ml
netrin-1 50 ng/ml
netrin-1 100 ng/ml
netrin-1 150 ng/ml
netrin-1 200ng/ml
netrin-1 500ng/ml
netrin-1 1000ng/ml
E
Normalized TH surface area (μm 2)
control
netrin-1 (400 ng/ml)
F
6-OHDA
M.W (kDa)
CTRL
PBS
NTN1
DCC
Netrin-1
PTEN
PS6
B-actin
