# Supplementary figures and images for: Netrin‐1 and its receptor DCC modulate survival and death of dopamine neurons and Parkinson’s disease features
Source: EMBO J. 2020 Dec 22;40(3):e105537. doi: 10.15252/embj.2020105537 (PMC7849168; doi:10.15252/embj.2020105537)

Supple Figure 2

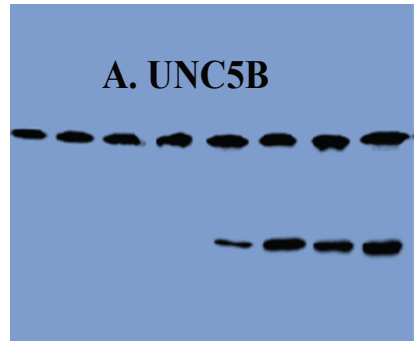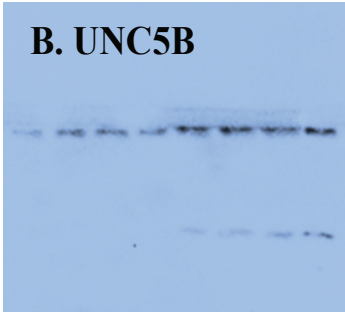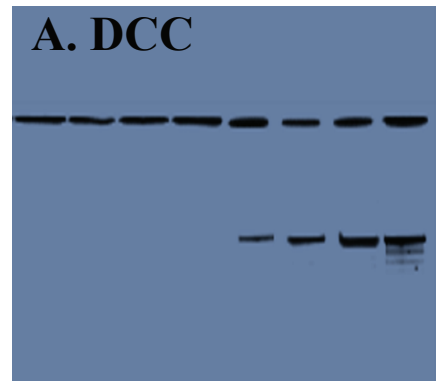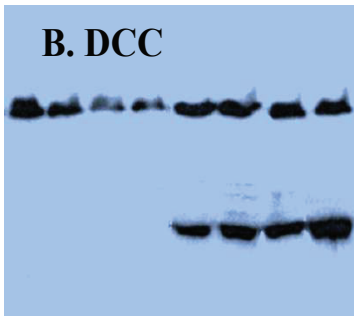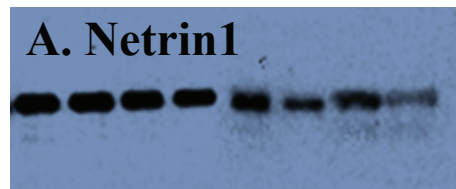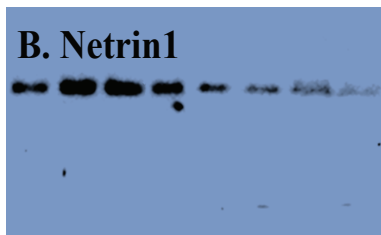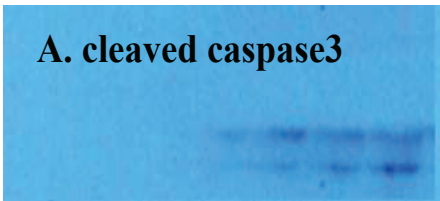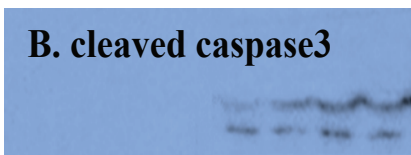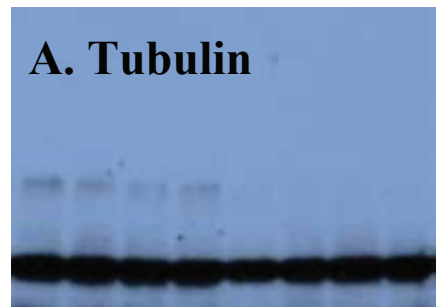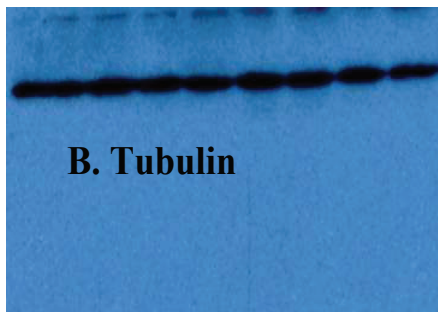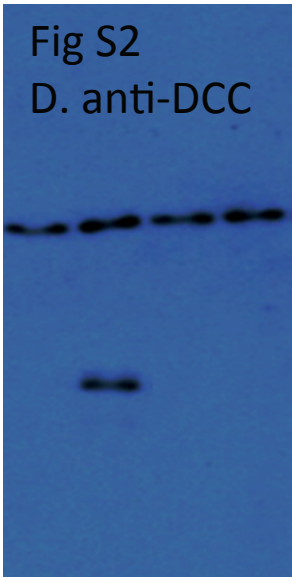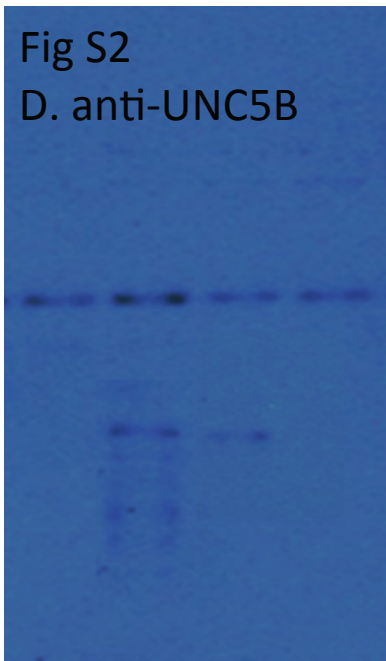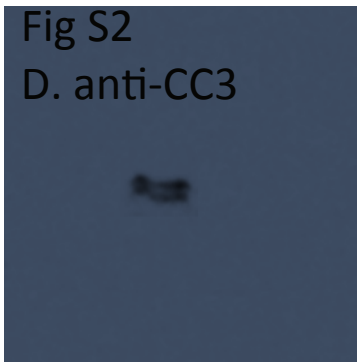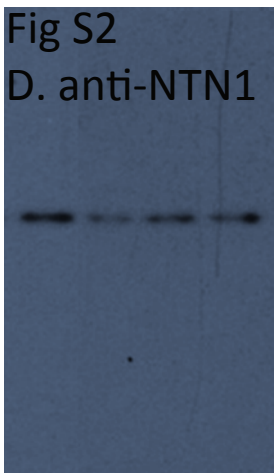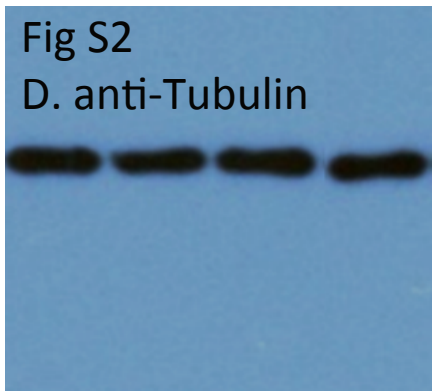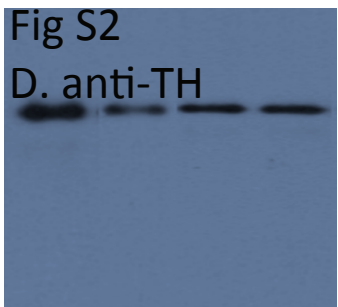

Supplement: Supplementary file 2 — Source Data for Expanded View/Appendix [file EMBJ-40-e105537-s005.zip › Appendix_Figure_Source_data/Appendix_FigS2_source_data.pdf]

Appendix Figure S3

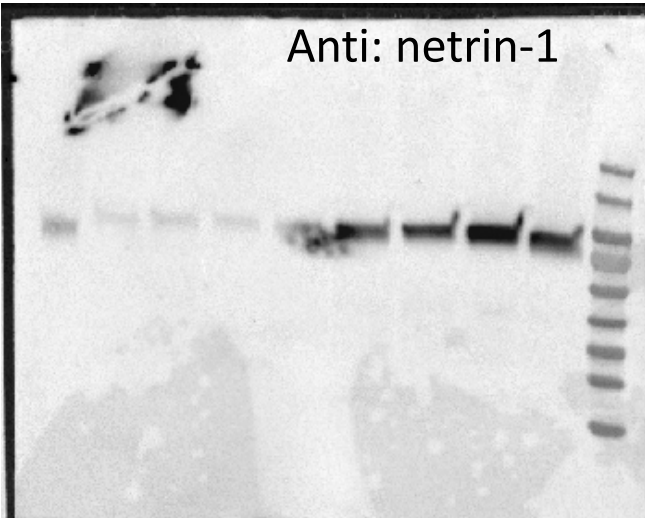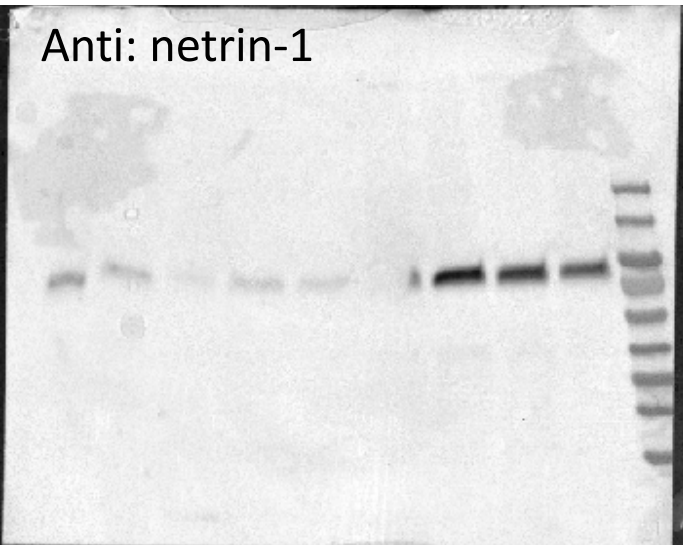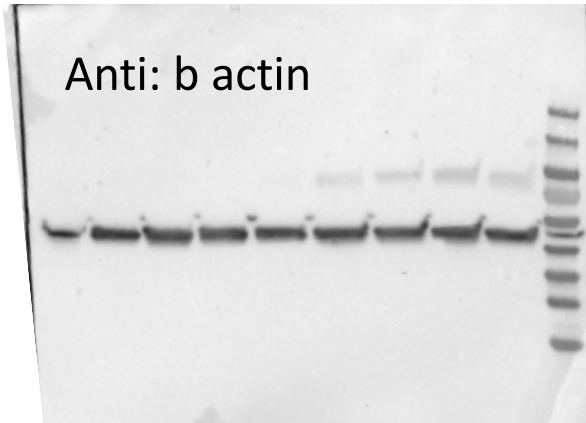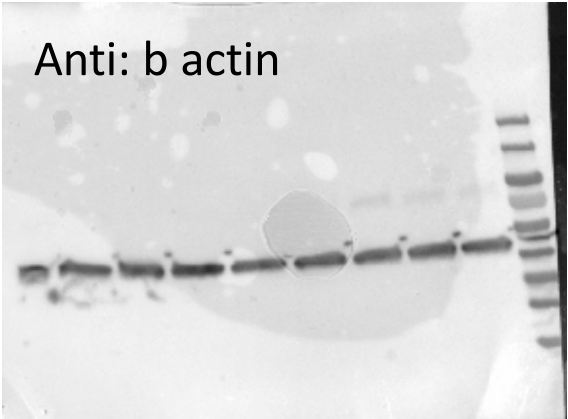

Supplement: Supplementary file 2 — Source Data for Expanded View/Appendix [file EMBJ-40-e105537-s005.zip › Appendix_Figure_Source_data/Appendix_FigS3_source_data.pdf]

Appendix Figure S4

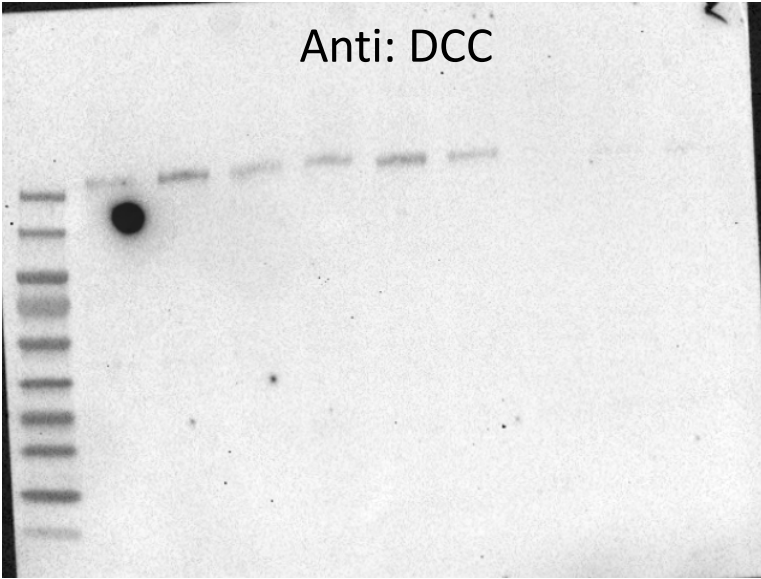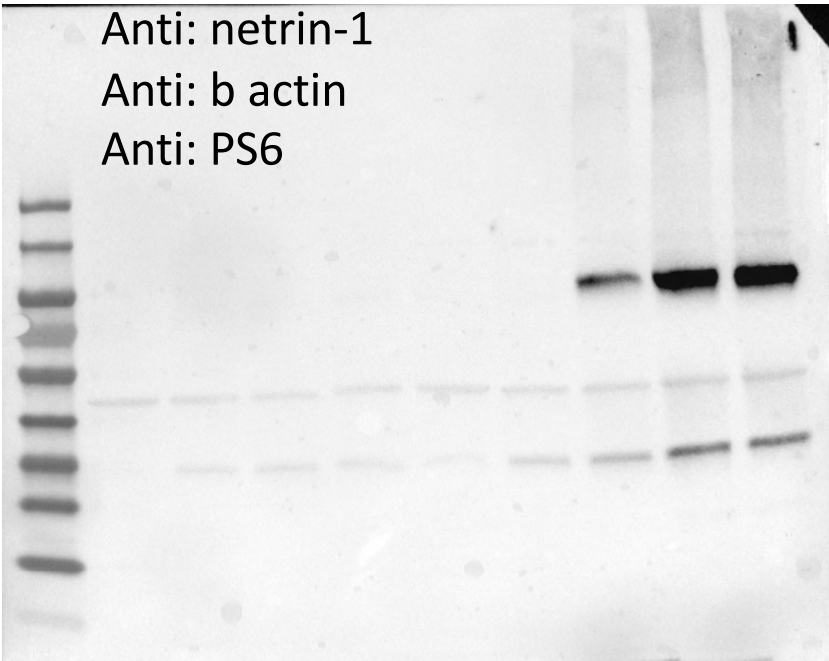

Anti: PTEN

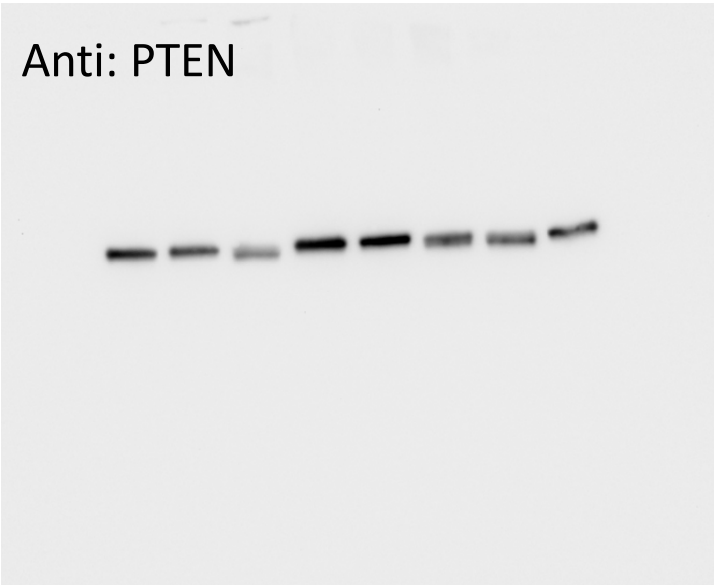

Supplement: Supplementary file 2 — Source Data for Expanded View/Appendix [file EMBJ-40-e105537-s005.zip › Appendix_Figure_Source_data/Appendix_FigS4_source_data.pdf]

Figure 2F

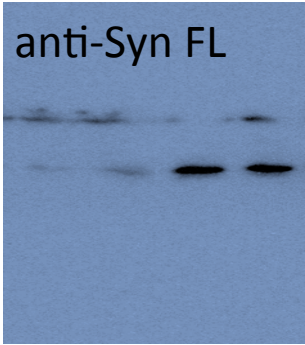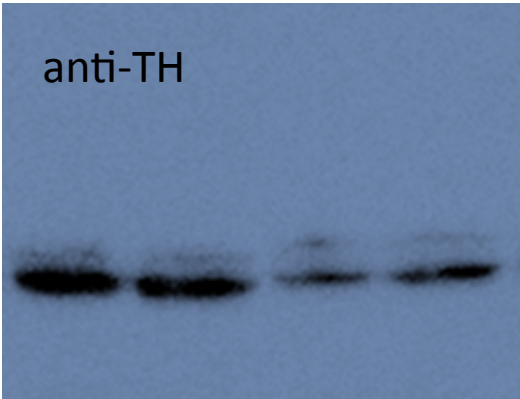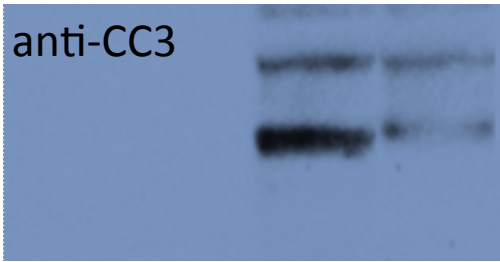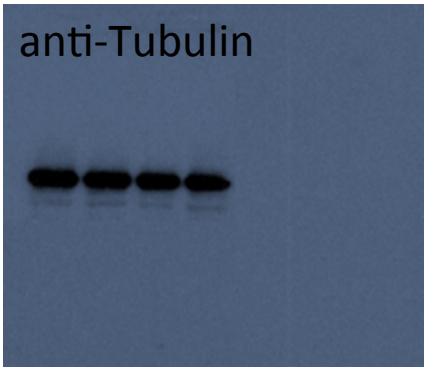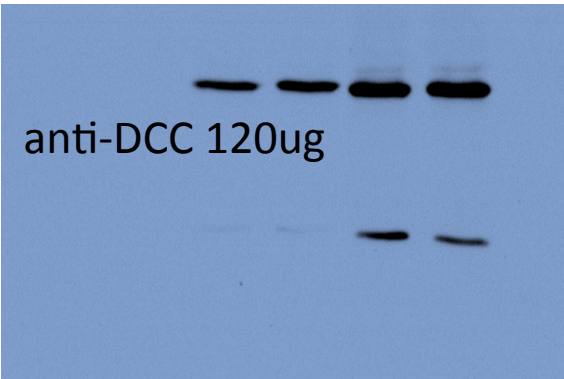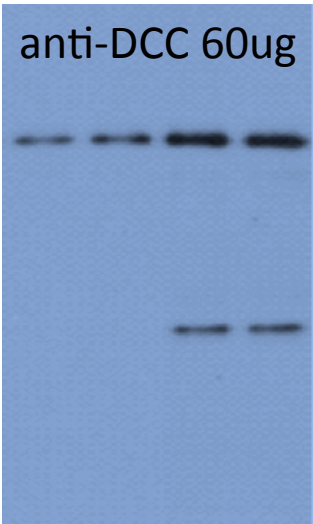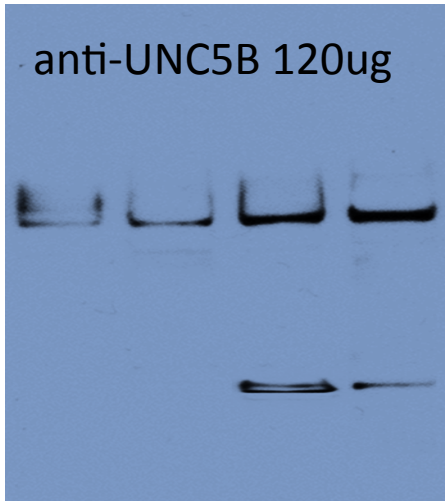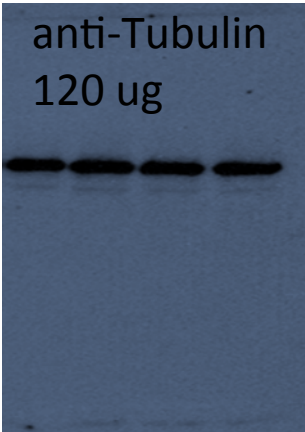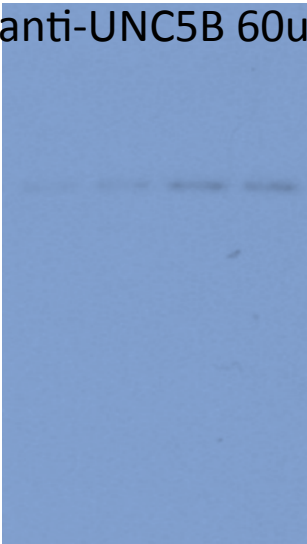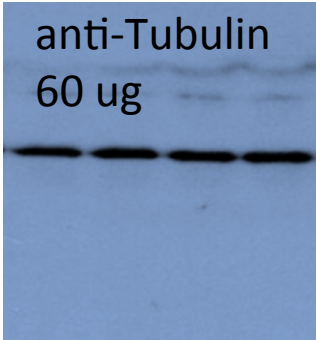

Supplement: Supplementary file 4 — Source Data for Figure 2 [file EMBJ-40-e105537-s002.pdf]

Figure 3D

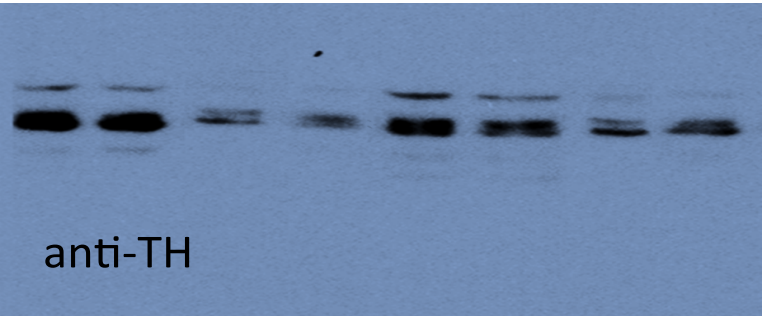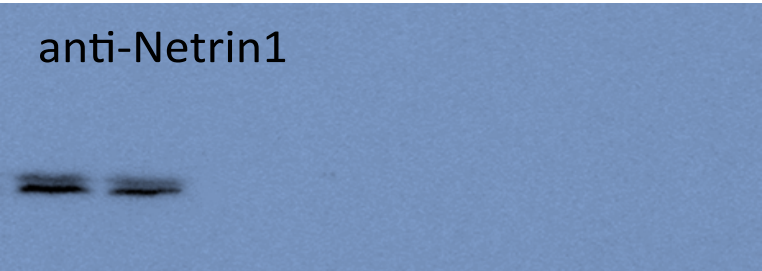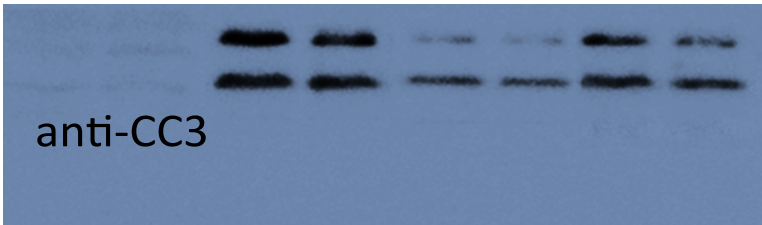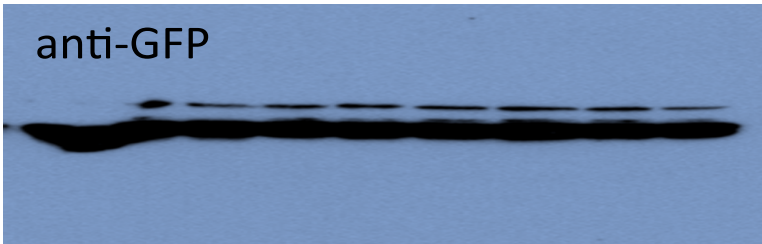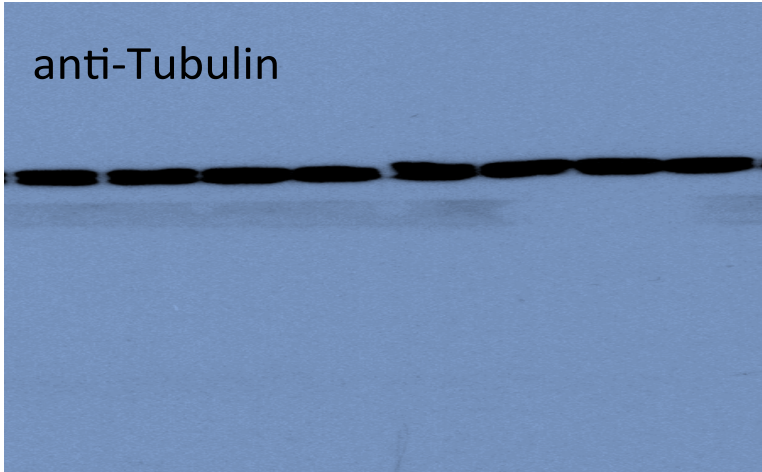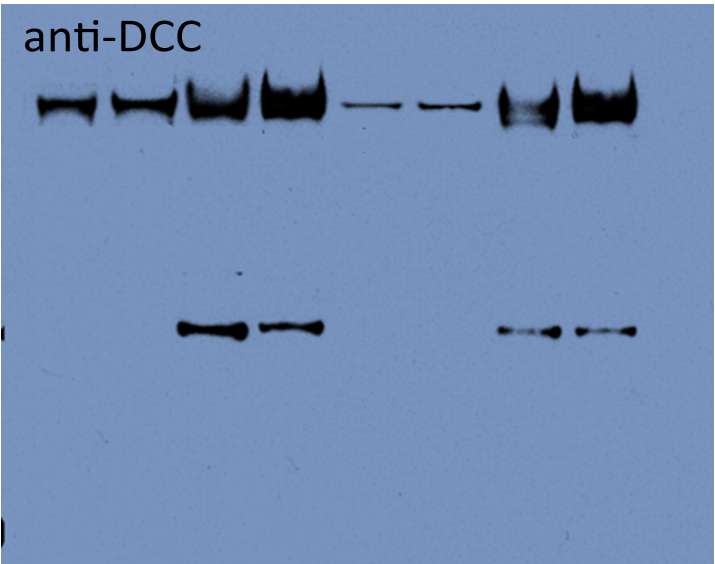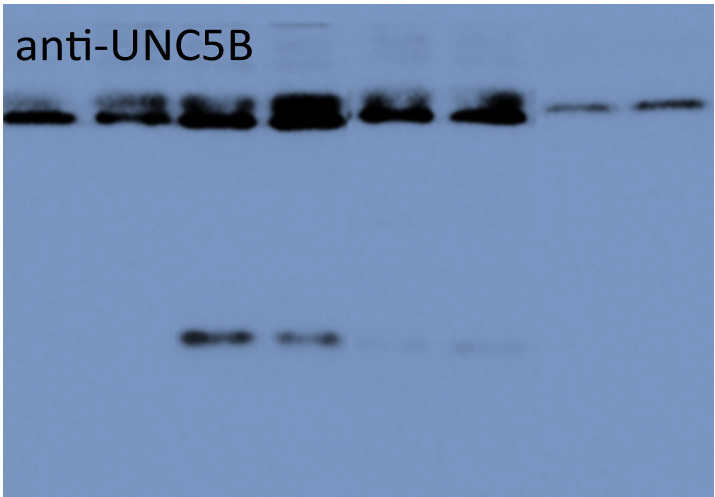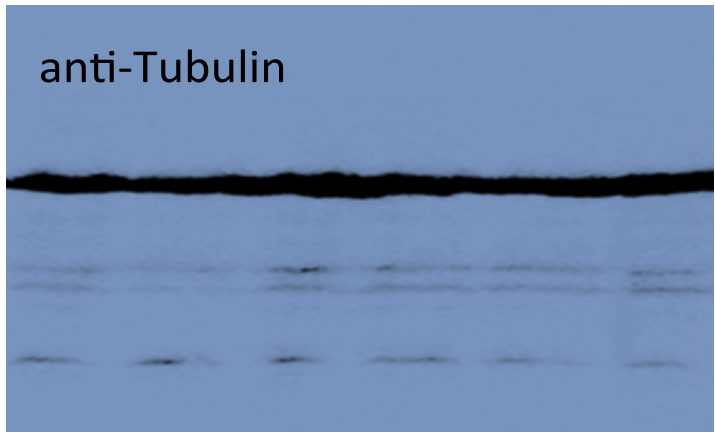

Supplement: Supplementary file 5 — Source Data for Figure 3 [file EMBJ-40-e105537-s003.pdf]

Figure 6B

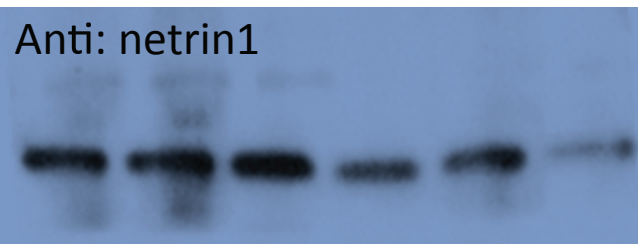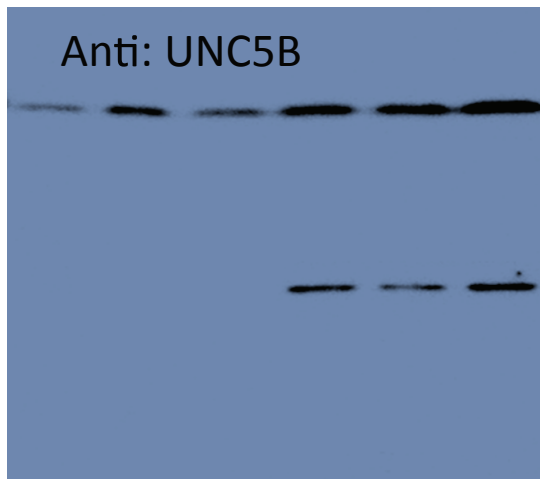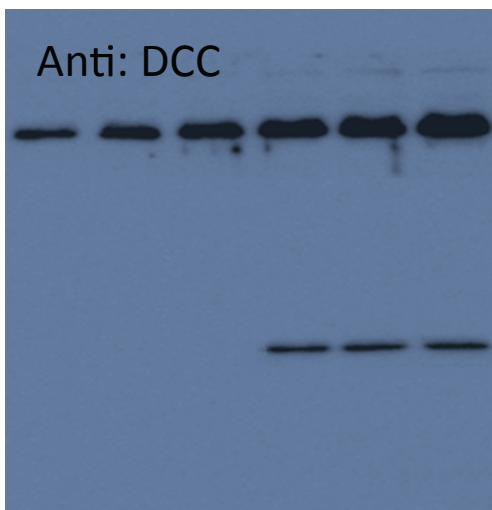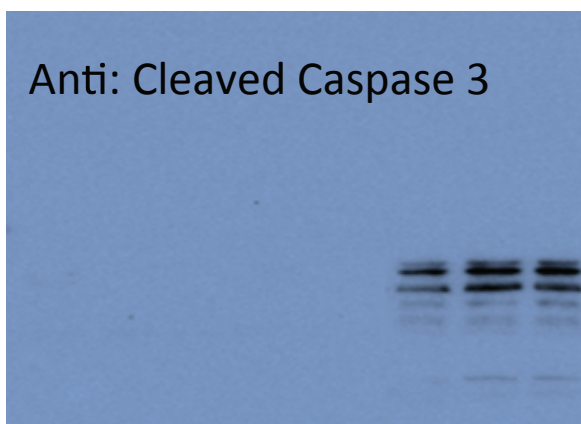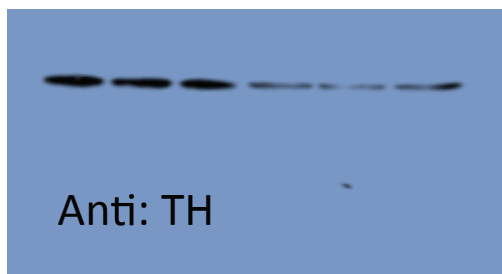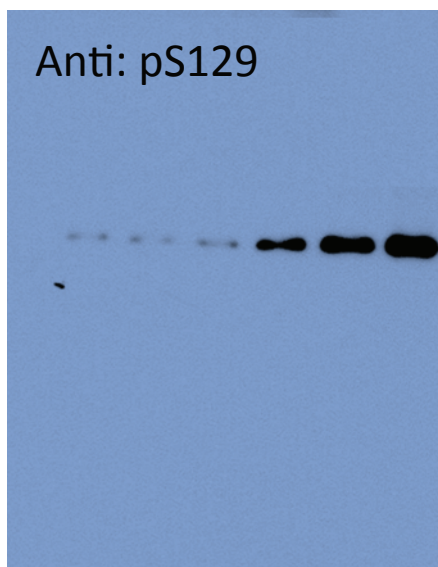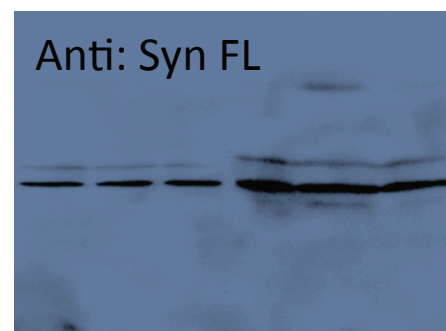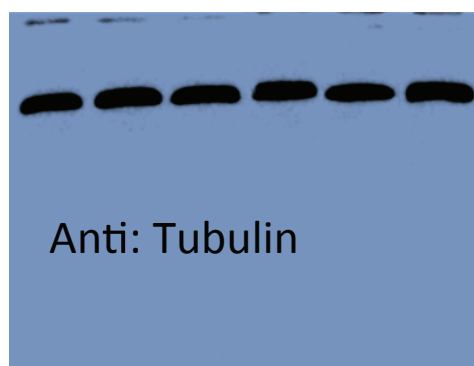

Supplement: Supplementary file 6 — Source Data for Figure 6 [file EMBJ-40-e105537-s004.pdf]
